# The Influence of Intense Chemical Pollution on the Community Composition, Diversity and Abundance of Anammox Bacteria in the Jiaojiang Estuary (China)

**DOI:** 10.1371/journal.pone.0033826

**Published:** 2012-03-21

**Authors:** Baolan Hu, Lidong Shen, Ping Du, Ping Zheng, Xiangyang Xu, Jiangning Zeng

**Affiliations:** 1 Department of Environmental Engineering, Zhejiang University, Hangzhou, China; 2 Department of Ecology and Environment, Second Institute of Oceanography, State Oceanic Administration, Hangzhou, China; National Institute of Water & Atmospheric Research, New Zealand

## Abstract

Continuous chemical pollution is one of the most serious environmental problems in the Jiaojiang Estuary of the East Sea (China). This chemical pollution has significantly changed the estuarine environmental conditions and may have profoundly influenced the distribution of anammox bacterial communities in this estuary. Here, we investigated the influence of chemical pollution on the community composition, diversity and abundance of anammox bacteria in Jiaojiang estuarine sediments. Phylogenetic analysis of 16S rRNA genes showed that the majority of anammox bacterial sequences retrieved from the estuarine intertidal sediments were associated with *Kuenenia*. In contrast, different anammox communities composed of *Brocadia*, *Kuenenia*, *Scalindua* and *Jettenia* were found in the estuarine subtidal sediments. Redundancy analysis (RDA) indicated that the sediment nitrobenzene and organic content had significant impacts on the distribution of anammox communities in the intertidal sediments. Pearson correlation analysis showed that the diversity of anammox bacteria in the intertidal sediments was positively correlated with the organic content. In contrast, RDA results showed that the nitrobenzene content, NO_3_^−^ concentration and salinity significantly influenced the distribution of anammox communities in the subtidal sediments. The diversity and relative abundance of anammox bacteria in the subtidal sediments were positively correlated with NO_3_^−^ concentration.

## Introduction

The process of anammox (anaerobic ammonium oxidation), which refers to the oxidation of ammonium coupled with the reduction of nitrite under anaerobic conditions, has been predicted to be a more thermodynamically favourable process than aerobic ammonium oxidation [1]. However, the anammox process had not been confirmed until its discovery in a wastewater treatment plant in the Netherlands [2]. Subsequently, the anammox process has been reported to be a new sink for fixed nitrogen in various natural ecosystems [3]–[5]. The relative contribution of anammox to total N_2_ fluxes has been reported to be 20–79% in anoxic marine sediments [6]–[8]. Anammox bacteria are also mainly responsible for nitrogen loss in anoxic marine water columns, especially in oxygen minimum zones (OMZ) [9]–[11].

To date, the anammox process has been exclusively linked to one group of organisms: monophyletic members of the phylum *Planctomycetes* [12]–[15]. Members of the five described anammox bacterial genera (*Brocadia*, *Kuenenia*, *Scalindua*, *Anammoxoglobus* and *Jettenia*) have been detected in many different wastewater treatment systems [12]–[19], and *Scalindua* is ubiquitous in natural ecosystems [8], [20], [21]. The majority of available anammox bacterial 16S rRNA gene sequences from marine and freshwater environments are related to the *Scalindua* genus and exhibit surprisingly low diversity [8], [21], [22].

An estuarine ecosystem is a partially enclosed coastal body of water with one or more inflowing rivers or streams and a free connection to the open sea. The rivers or streams can potentially bring a large amount of pollutants from point and non-point sources into an estuary, which would result in highly polluted estuaries. In particular, estuaries are recognised as the site of significant bacterial removal of anthropogenically derived inorganic nitrogen [23]. Previous studies have investigated the distribution of anammox bacteria in several estuarine ecosystems [23]–[26]. Unlike most marine and freshwater environments, where only members of the *Scalindua* genus have been detected, different anammox communities composed of *Brocadia*, *Kuenenia*, *Scalindua* and *Jettenia* have been found in the Cape Fear River Estuary [25]. In addition, variations in the contributions of anammox to local or regional estuarine nitrogen loss have been reported [24]–[26] and have likely been due to distinct environmental conditions that may influence the composition, abundance and distribution of anammox bacteria in different estuarine ecosystems. Anthropogenic disturbances of variable sources may further complicate the anammox bacteria-environment relationship in estuarine ecosystems [27]. To date, the interactions of anammox bacteria with different environmental factors and anthropogenic disturbances are still poorly unknown in estuarine environments. Many researchers have found it helpful to employ diversity and abundance measurements for understanding both the microbial diversity and the ecology of anammox bacteria in estuarine environments and to ascertain the influences of different environmental factors on anammox community structures [25], [27].

The Jiaojiang Estuary is located in one of the most economically developed areas in China where many chemical plants are found. Because the Jiaojiang Estuary has been seriously polluted by chemical industry over a long period of time (since 1980s) [28], the entire estuary has been polluted by the chemicals and has significantly influenced the distribution of macrozoobenthos, benthic polychaetes and zooplankton within this estuary [28]–[30]. Chemical pollution has significantly changed the estuarine environmental conditions and may also have profoundly influenced the distribution of anammox communities within this estuary.

The primary objectives of this study were to determine the community composition, diversity and abundance of anammox bacteria in the chemically polluted Jiaojiang Estuary of the East Sea (China) and to ascertain the influences of chemical pollution on the distribution of anammox communities in the intertidal and subtidal sediments.

## Materials and Methods

### Ethics Statement

No specific permits were required for the described field studies.

### Site description and sample collection

**Figure 1:**
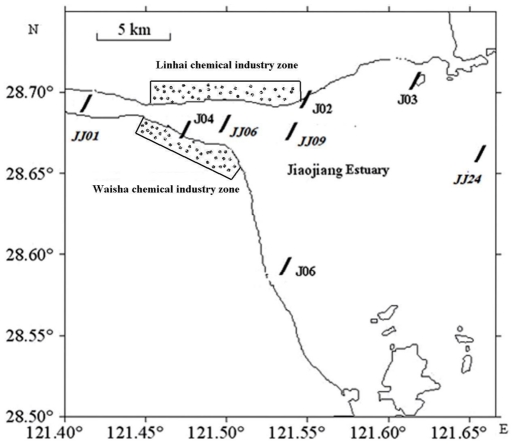
Map of the sampling sites within the Jiaojiang Estuary. The subtidal sediment sampling sites are indicated with italics.

The Jiaojiang Estuary (28°50′N to 28°75′N and 121°40′E to 121°68′E) is approximately 35 km long with a watershed area of 6750 km^2^. Within the estuary, the average width of the channel is approximately 1.2 km with a maximum width of 1.8 km at the mouth (Figure 1). The Jiaojiang Estuary is a partially enclosed coastal body of water with many inflowing rivers (such as the Ling River and Yongning River) and a connection to the East Sea of China. More than 3000 chemical plants are distributed along both sides of the estuary, which form the Waisha (on the south side) and Linhai (on the north side) chemical industry zones (Figure 1). The wastewater from these chemical industry zones contains large amounts of organic matter, chemicals (e.g. polycyclic aromatic hydrocarbon (PAH), aniline and nitrobenzene) and dissolved inorganic nitrogen, which are released into the Jiaojiang Estuary. This discharge has resulted in the estuary that is hypernutrified and highly polluted by chemicals.

Sediment samples were collected in October 2009 using box cores from 8 sampling sites along the Jiaojiang Estuary (Figure 1), and the uppermost 3 cm of sediment was carefully collected. The samples (J02, J03, J04 and J06) were collected from the estuarine mid-intertidal zone where the sediment is regularly exposed and submerged by tides and is significantly influenced by chemical pollution due to the close proximity to the chemical industry zones. To further investigate the influence of chemical pollution on the distribution of anammox communities in the Jiaojiang Estuary, four subtidal sediment samples were also collected and analysed. The samples (JJ01, JJ06, JJ09 and JJ24) were collected from the subtidal zone where the sediment is continuously submerged by the water column and is less influenced by chemical pollution relative to the intertidal sediments. The sediment samples were immediately transferred on ice to the laboratory and stored at −80°C until the DNA was extracted. The basic physical and chemical parameters of the estuarine overlying water are shown in Table 1. The basic physical and chemical parameters of the estuarine sediments are shown in Table 2.

**Table 1:** The basic physical and chemical parameters of the overlying water of the Jiaojiang Estuary.

Sample	Water depth(m)	Temperature(°C)	#NH_4_^+^-N(µM)	NO_2_^—^N(µM)	NO_3_^—^N(µM)	DO(µM)	PO_4_^3—^P(µM)	Salinity(ppt)
JJ01	7	22.1	4.3	0.6	133.0	241.9	6.5	11.9
JJ06	6	21.6	4.6	0.9	97.2	229.4	3.4	20.3
JJ09	8	21.7	1. 2	0.6	83.4	228.1	2.5	21.7
JJ24	9	22.6	2.0	0.3	42.8	230.3	2.5	25.2

**Table 2:** The basic physical and chemical parameters of the Jiaojiang estuarine sediments.

Samples	Organic carbon(%)	Sulfide(%)	Eh(mV)	PAH(10^−9^)	Aniline(10^−9^)	Nitrobenzene (10^−9^)
J02	0.6	0.6	−197.0	99.3	78.0	284.0
J03	1.6	0.4	−293.0	108.9	81.0	321.0
J04	0.5	2.2	−260.0	85.9	43.0	267.0
J06	1.0	1.6	−130.0	106.5	90.0	223.0
JJ01	1.2	31.2	−92.0	164.7	215.0	52.0
JJ06	1.3	1.5	−126.0	142.2	250.0	72.0
JJ09	0.9	3.9	−153.0	76.2	206.0	88.0
JJ24	0.8	0.5	−155.0	124.7	262.0	129.0

### DNA extraction and PCR amplification

DNA was extracted using a Power Soil DNA kit (Mo Bio Laboratories, Carlsbad, California, USA) according to the manufacturer's instructions. The extracted DNA was examined by electrophoresis in a 1.0% agarose gel.

Different combinations of primers (Table 3) were used for the amplification of anammox bacterial 16S rRNA genes. A nested PCR approach was chosen based on its amplification yield and without nonspecific PCR products with positive controls. In the first round of PCR, *Planctomycetales* 16S rRNA genes were amplified with Pla46f [31] as the forward primer and 1545r [32] as the reverse primer. In the second round, the reaction was performed using the anammox bacteria-specific primers, Amx368f [18] and Amx820r [17]. The PCR reaction mixture (25 µl in total) contained 2.5 µl 10×PCR buffer (containing 2 mM MgCl_2_), 20 mM of each deoxyribonucleoside triphosphate, 1 mM of each primer, 1 U of *Taq* polymerase and 1 µl of DNA template (1–10 ng). The PCR thermal cycle programs were performed as previously described [33]. The amplified products (478 bp) were examined by electrophoresis in a 1.0% agarose gel.

**Table 3:** Primers used for PCR in this study.

Primers	Sequence 5′-3′	Specificity	*E. coli* position number	Reference
Pla46f	GGATTAGGCATGCAAGTC	*Planctomycetes*	46–63	[31]
1545r	CAKAAAGGAGGTGATCC	All bacteria	1529–1545	[32]
Amx368f	TTCGCAATGCCCGAAAGG	anammox bacteria	368–385	[18]
Amx820r	AAAACCCCTCTACTTAGTGCCC	*Brocadia*/*Kuenenia*	820–841	[17]
Amx694f	GGGGAGAGTGGAACTTCGG	Anammox bacteria	694–713	[34]
Amx960r	GCTCGCACAAGCGGTGGAGC	Anammox bacteria	960–979	[34]

### Cloning and sequencing

The PCR products were cloned using the pMD19-T vector (TaKaRa, Bio Inc., Shiga, Japan) according to the manufacturer's instructions. Plasmid DNA was isolated using the Gene JET™ Plasmid Miniprep kit (Fermentas Life Sciences, Burlington, Canada) and was digested with 5 U of EcoRI enzyme in EcoRI buffer for 1.5 h at 37°C. The digestion products were examined for an insert of the expected size using agarose (1.0%) gel electrophoresis. At least 30 inserts from each sample were subjected to sequence analysis using an ABI3100 automated sequencer (Applied Biosystems, California, USA).

### Phylogenetic analysis

Phylogenetic analysis of the sequences was conducted using ARB software as previously described [18]. Phylogenetic analysis was performed with the neighbour-joining method with 50% sequence conservation filters for *Planctomycetes*. Bootstrap analysis with 1,000 replicates was applied to assign confidence levels to the nodes of the tree.

### Real-time quantitative PCR (qPCR)

A primer set (Amx694f-Amx960r) (Table 3) targeting 16S rRNA genes was used to compare the relative abundances of anammox bacteria in different sediment samples collected from the Jiaojiang Estuary. The qPCR analysis was performed using an iCycler iQ5 thermocycler and real-time detection system (Bio-Rad, California, USA). The relative anammox bacterial DNA concentration in each sample was determined by identifying its cycle threshold (C_T_). The qPCR was conducted as previously described [34]. The specificity of the PCR amplification was determined by the melting curve and the amplified products (285 bp) by gel electrophoresis.

### Statistical analyses

Operational taxonomic units (OTUs) for the determination of 16S rRNA gene diversity of anammox bacteria were defined using 3% differences in the nucleotide sequences, as determined by using the furthest neighbour algorithm in the DOTUR program [35]. DOTUR was also used to generate Chao and Shannon indexes for each clone library. The coverage of clone libraries was calculated as follows: *C* = (1−[*n*_1_ */N*])×100, where *n*_1_ is the number of unique OTUs, and *N* is the total number of clones in a library. The ecological distribution of anammox communities and their correlations with environmental factors were determined using principal components analysis (PCA) and redundancy analysis (RDA), respectively, using the software CANOCO [36]. The Hellinger transformation [37] of the obtained data was applied before the PCA and RDA tests. In addition, Pearson correlation analysis and partial correlation analysis (significance level α = 0.05) were used to test for correlations between the anammox bacterial diversity, relative abundance and different environmental factors.

### Nucleotide sequence accession numbers

The sequences obtained in this study are available in GenBank under accession numbers JN051510-JN051644.

## Results and Discussion

### The influences of chemical pollution on the community composition, diversity and relative abundance of anammox bacteria in Jiaojiang estuarine intertidal sediments

**Figure 2:**
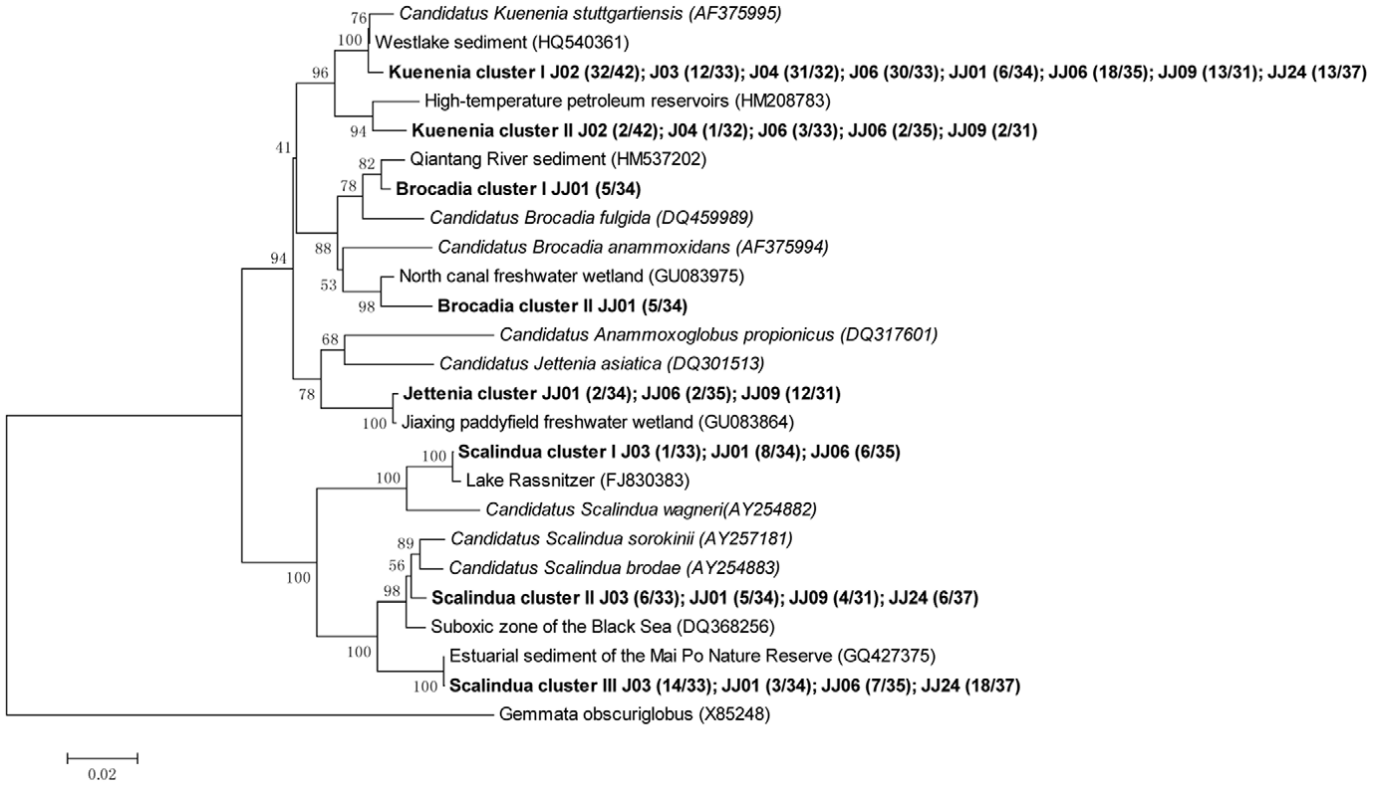
Phylogenetic tree showing the relationships between anammox bacteria-related 16S rRNA gene fragments in the clone libraries of samples from the Jiaojiang Estuary (bold) and known anammox bacteria (italics). Clone names are composed as follows: the sample name followed by the number of times a sequence was detected among all of the tested clones of a sample.

**Figure 3:**
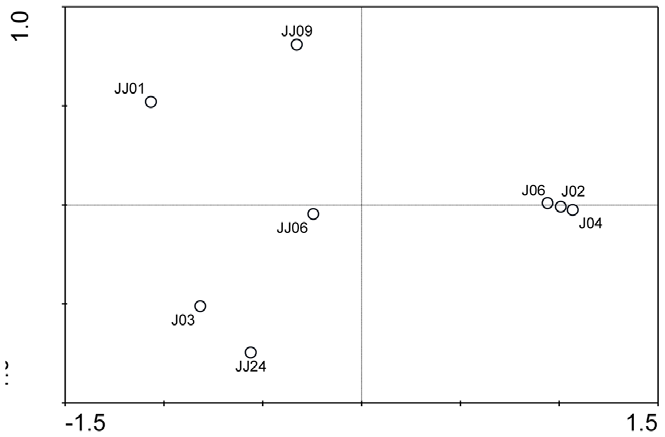
PCA ordination diagram of the anammox communities calculated using 16S rRNA gene sequences from Jiaojiang estuarine sediments.

**Figure 4:**
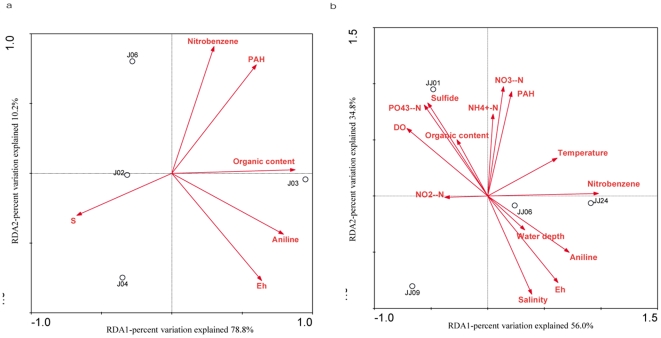
RDA ordination plots for the first dimensions showing the relationships between the anammox communities and environmental factors within the estuarine intertidal (a) and subtidal sediments (b). Correlations between environmental factors and RDA axes are represented by the length and angle of the arrows.

A total of 137 gene sequences based on the nested PCR amplification of 16S rRNA genes were obtained from four different samples collected from Jiaojiang estuarine intertidal sediments. Cloning and sequence analysis confirmed that 132 (96.4%) of the amplified sequences were related to known anammox genera. The phylogenetic analysis of 16S rRNA genes showed that two anammox genera, *Kuenenia* (*Kuenenia* cluster I and II) and *Scalindua* (*Scalindua* cluster I, II and III), were detected in the intertidal sediments and that *Kuenenia* bacteria appeared to be the most common species (Figure 2). The sequence identity of each anammox cluster and the corresponding anammox bacteria are provided in Table 4. PCA results showed that the anammox communities of samples J02, J04 and J06 were highly similar. In contrast, the community of sample J03 was distant from those of the remaining intertidal sediment samples (Figure 3). The varying anammox community compositions found in the intertidal sediments may be related to the different environmental conditions at the different sampling sites. The RDA results showed that the nitrobenzene and organic content of the sediment appeared to have the most significant correlation with the distribution of the anammox communities in the intertidal sediments (p<0.05) (Figure 4a). Furthermore, the different anammox communities found in different intertidal sediment samples may also be related to the sampling location. Because the sampling site J03 was located near the mouth of the Jiaojiang Estuary (Figure 1), the anammox communities could be significantly influenced by the open sea. This may also contribute to the presence of *Scalindua* bacteria in sample J03.

**Table 4:** Sequence matches between each cluster and known anammox bacteria.

Anammox bacterial cluster	Related anammox bacteria	Sequence identity (%)
*Kuenenia* cluster I	*Candidatus Kuenenia stuttgartiensis*	97.2–99.2
*Kuenenia* cluster II	*Candidatus Kuenenia stuttgartiensis*	95.0–96.1
*Brocadia* cluster I	*Candidatus Brocadia fulgida Candidatus Brocadia anammoxidans*	96.6–97.5
*Brocadia* cluster II	*Candidatus Scalindua wagneri Candidatus Scalindua brodae*	93.0–93.9
*Scalindua* cluster I	*Candidatus Scalindua brodae Candidatus Jettenia asiatica*	94.0–96.0
*Scalindua* cluster II		95.4–98.1
*Scalindua* cluster III		92.2–94.9
*Jettenia* cluster		92.6–94.5

The diversity of anammox bacterial sequences in each sample was compared based on the numbers of OTUs, the Shannon-Wiener index and the *S*_chao1_ estimators (Table 5). The overall anammox diversity was low within the intertidal sediments. The diversity of anammox bacteria in sample J03 was higher than that in the remaining intertidal sediment samples (Table 5). Pearson correlation analysis showed that the sediment organic content significantly influenced the diversity of anammox bacteria within the intertidal sediments (Table 6).

**Table 5:** Summary of the anammox bacterial diversity within the Jiaojiang Estuary.

Samples	No. of sequences	Coverage	No. of OTUs^1^	Shannon-Weiner^2^	Chao1^2^
**Intertidal sediments**	132	0.96	10	0.80	19.00
J02	34	1.00	1	0	1.00
J03	33	0.91	9	1.86	15.00
J04	32	1.00	1	0	1.00
J06	33	0.97	2	0.14	2.00
**Subtidal sediments**	137	0.97	21	2.07	21.75
JJ01	34	0.91	10	2.02	12.00
JJ06	35	0.94	9	1.81	9.33
JJ09	31	0.97	7	1.66	7.00
JJ24	37	0.95	5	1.07	5.50

1 No. of OTUs were determined using the DOTUR program based on the 3% sequence difference.

2 The coverage, Shannon-Weiner and *S*_Chao1_ richness estimators were calculated using the OTUs data.

**Table 6:** Correlation analysis of environmental factors and anammox bacterial diversity and abundance within the Jiaojiang Estuary.

Environmental factors	Pearson correlation coefficient			
	No. of OTUs	Shannon index	Chao1	Relative abundance
**Intertidal sediments**				
Organic content	0.9353	0.9170	0.9595	−0.4118
Sulfide	−0.6102	−0.6188	−0.6194	−0.3370
Eh	−0.5920	−0.6272	−0.6298	−0.5306
PAH	0.6373	0.6088	0.6065	−0.4465
Aniline	0.3387	0.3066	0.3041	−0.4449
Nitrobenzene	0.6947	0.7286	0.7310	0.6475
**Subtidal sediments**				
Temperature	−0.5621	−0.6799	−0.3439	−0.6187
NH_4_^+^-N	0.8066	0.6320	0.8085	0.5876
NO_2_^−^-N	0.7365	0.7410	0.5507	0.6620
NO_3_^−^-N	**0.9698**	**0.9724**	**0.9695**	**0.9843**
DO	0.6273	0.5396	0.8007	0.6025
PO_4_^3−^-P	0.8019	0.7207	0.9251	0.7636
Salinity	−0.8909	**−0.9578**	**−0.9636**	**−0.9472**
Organic content	0.9157	0.8209	0.8421	0.7667
Sulfide	0.6938	0.6580	0.8368	0.7296
Eh	0.9004	0.8022	0.8728	0.8193
PAH	0.5934	0.3747	0.7115	0.9233
Aniline	−0.4389	−0.6372	−0.4150	−0.6928
Nitrobenzene	**−0.9835**	**−0.9952**	**−0.9441**	**−0.9915**

Boldface denotes a p value of <0.05, which is typically regarded as significant, as determined by SPSS version 15.0 program (SPSS, Chicago, Illinois, USA).

**Figure 5:**
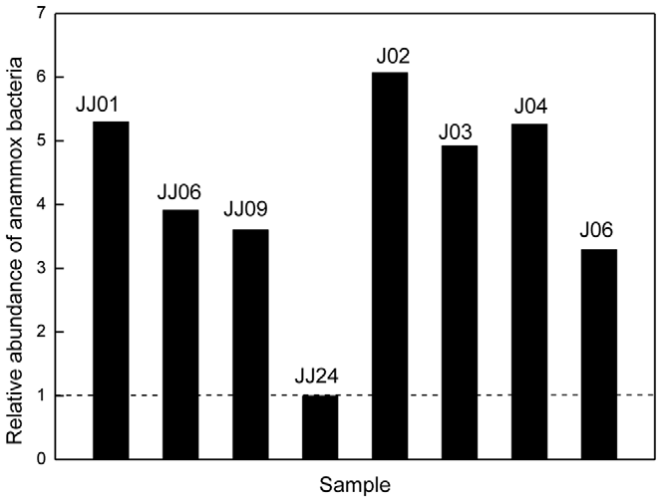
Relative abundances of anammox bacteria in different samples from the Jiaojiang Estuary.

The relative abundance of anammox bacteria in each sample was compared based on the quantification of anammox 16S rRNA genes using a qPCR method, as previously described [34]. Here, the sample that had the lowest anammox PCR product concentration was used as the baseline (i.e., the relative abundance of anammox bacteria was recorded as “1.0”) (Figure 5). The qPCR results showed a heterogeneous distribution of anammox bacterial abundances within the intertidal sediments. As shown in Figure 5, the relative abundance of anammox bacteria in the intertidal sediments decreased along the estuary. However, the relative abundance of anammox bacteria in the intertidal sediments did not show a significant correlation with any of the environmental factors investigated in the present study (Table 6).

Here, three typical chemical pollutants, including PAH, aniline and nitrobenzene that were derived from the chemical industry zones, were selected to investigate their impacts on distribution of anammox communities in Jiaojiang estuarine sediments. Among the three chemical pollutants, nitrobenzene was found to contribute significantly to the spatial distribution of anammox communities within Jiaojiang estuarine intertidal sediments. Nitrobenzene is frequently released into the environment via the effluent from chemical plants and has a high potential for pollution in the environment. Nitrobenzene is extremely recalcitrant to enzymatic attack due to the stability of the benzene ring caused by the electron-withdrawing nature of the nitro group [38]. Owing to its stability, persistence and toxicity, nitrobenzene has been listed as a priority pollutant by the United States Environmental Protection Agency [39]. Li et al. [40] have reported that nitrobenzene significantly influenced the bacterial communities in Songhua River sediments in Northeastern China following a nitrobenzene pollution event and that nitrobenzene led to a clear decrease in the bacterial diversity. Furthermore, Zhao et al. [38] have reported that nitrobenzene significantly influenced nitrification rates and communities of ammonium-oxidizing bacteria in nitrobenzene-contaminated soil. In the present study, the nitrobenzene was found to have great impact on the distribution of anammox communities and resulted in the low diversity of anammox bacteria in the intertidal sediments of the Jiaojiang Estuary. In contrast to reports on the distribution of anammox bacteria in estuarine intertidal sediments, where different anammox genera occur together [24], [25], [41], we detected only one anammox genus in Jiaojiang intertidal sediments (except for sampling site J03) and observed a diversity that was clearly lower than that in previous reports. The major difference between our estuary and the reported estuaries was the intense chemical pollution (especially nitrobenzene pollution) in the Jiaojiang Estuary [28], [29], [42]. This indicated the adverse effect of nitrobenzene on the distribution of anammox communities in intertidal sediments. Because nitrobenzene has a toxic effect on the growth of most microorganisms (autotrophic bacteria in particular), anammox bacteria may be acutely susceptible to nitrobenzene due to the autotrophy of these bacteria; therefore, it is likely that only certain anammox species that could tolerate high nitrobenzene contents could survive. It is well known that *Scalindua* genus was the dominant anammox species in the reported estuaries [24], [25], [41]. However, *Kuenenia*, rather than *Scalindua*, appeared to be the most common representatives in Jiaojiang intertidal sediments. This result suggested that the chemical pollution significantly changed the estuarine environmental conditions and may be an important factor controlling the distribution of anammox bacteria in the intertidal sediments. The wide distribution of *Kuenenia* bacteria in the intertidal sediments may indicate their greater ability to cope with nitrobenzene. The genome of the *Candidatus Kuenenia stuttgartiensis* was assembled from a complex microbial community grown in a sequencing batch reactor (74% enriched in this bacterium) using a metagenomics approach. The assembled genome showed that this bacterium has very many multidrug export proteins in its genome [43] which may help in coping with the nitrobenzene.

The organic content also significantly contributed to the spatial variations in the anammox community composition and diversity within the intertidal sediments. A higher organic content can lead to a higher NO_3_^−^ reduction rate, which may lead to a greater release of NO_2_^−^ and, thus, increase the level of NO_2_^−^ available to anammox bacteria. Furthermore, more NH_4_^+^ can be released through the process of mineralisation or dissimilatory NO_3_^−^ reduction using organic matter as an electron donor in environments with relatively high organic contents [11]. Higher levels of NH_4_^+^ and NO_2_^−^ would be available for the distribution of diverse anammox bacteria under conditions of relatively high organic contents. Estuaries are sinks for organic matter entering both from their catchments and also from the adjacent lands and urban areas. Various studies have shown that estuarine sediment organic contents are significantly influenced by anthropogenic inputs [27], [44]. In the present study, wastewater containing large amounts of organic matter [42] from nearby chemical industry may have provided an important source of organic matter to the estuarine sediments. Therefore, the influence of the organic content on the distribution of the anammox communities in the intertidal sediments may be related to the intense chemical pollution in the Jiaojiang Estuary.

### The influences of chemical pollution on the community composition, diversity and relative abundance of anammox bacteria in Jiaojiang estuarine subtidal sediments

A total of 145 gene sequences based on nested PCR amplification of 16S rRNA genes were obtained from the four different sediment samples collected from the estuarine subtidal sediments. Cloning and sequence analyses confirmed that 137 (94.4%) of the amplified sequences were related to known anammox genera. The phylogenetic analysis of the 16S rRNA genes showed that different anammox communities composed of *Brocadia*, *Kuenenia*, *Scalindua* and *Jettenia* were found in the subtidal sediments and that *Kuenenia* and *Scalindua* appeared to be the most common anammox genera (Figure 2). Different anammox communities were observed in the different subtidal samples. The anammox communities of the four subtidal sediment samples were strikingly different from each other as shown in the PCA ordination diagram (Figure 3). Among the environmental factors investigated, the sediment nitrobenzene content, overlying water column NO_3_^−^ concentration and salinity contributed significantly to the distribution of the anammox communities within the subtidal sediments (p<0.05) (Figure 4b).

Differences in the anammox diversity and relative abundance were also observed in the different subtidal sediment samples. The diversity of the anammox bacteria decreased along the estuary (Table 5). Pearson correlation analysis showed that the diversity of anammox bacteria within the subtidal sediments was negatively correlated with the sediment nitrobenzene content and salinity and was positively correlated with NO_3_^−^ concentration (Table 6). Similar to the diversity, Pearson correlation analysis showed that the relative abundance of anammox bacteria in the subtidal sediments was negatively correlated with the sediment nitrobenzene content and salinity and was positively correlated with the overlying water column NO_3_^−^ concentration (Table 6). In fact, many of the parameters tested in our study are correlated. The NO_3_^−^ concentration and nitrobenzene content is significantly correlated (Pearson; r = −0.99, p = 0.012) and NO_3_^−^ concentration and salinity is also significantly correlated (Pearson; r = −0.96, p = 0.044). Therefore it is not reasonable to infer their (nitrobenzene content and salinity) individual causal effect on anammox diversity and abundance by Pearson correlation analysis along. Partial correlation analysis can be used to assess the extent to which a correlation between two parameters is due to their common relationship with a third variable [45]. We further applied partial correlation analysis in which the influence from NO_3_^−^ concentration was controlled to investigate the influence of salinity and nitrobenzene content on anammox diversity and abundance. The results showed that the correlation between the nitrobenzene content and anammox diversity or relative abundance was insignificant (p>0.05). The correlation between the salinity and anammox diversity or relative abundance was also insignificant (p>0.05). This indicated that part of the correlation between nitrobenzene or salinity with anammox diversity and relative abundance is attributable to the correlation with NO_3_^−^ concentration. From a statistical point of view, the observed effect of salinity and nitrobenzene on anammox diversity and abundance could probably be explained from the NO_3_^−^ data only. Thus the diversity and abundance of anammox bacteria in the estuarine subtidal sediments were mainly influenced by NO_3_^−^ concentration.

As observed for the estuarine intertidal sediments, RDA results showed that nitrobenzene contributed significantly to the spatial variation of anammox communities in the estuarine subtidal sediments, which further confirmed the strong influence of nitrobenzene on anammox bacterial distribution within the Jiaojiang Estuary. But when comparing the intertidal sediments where the anammox diversity was low, a higher overall anammox diversity was observed within the subtidal sediments where four different anammox genera were detected (Figure 2). As shown in Table 2, the nitrobenzene contents in the subtidal sediments were much lower than those in the intertidal sediments. This may indicate that nitrobenzene had a relatively weak influence on anammox communities in subtidal sediments, and thus lead to a higher diversity of anammox bacteria observed in the subtidal sediments. In addition, the physical environment is very different between the subtidal and the intertidal sediments: intertidal sediments are regularly exposed and inundated, while the subtidal sediments are continuously submerged. The difference in physical environment among those sediments may also contribute to the observed differences in anammox diversity.

In this study, RDA results showed that the salinity had great influence on the distribution of anammox communities in subtidal sediments. The salinity was also found to have significant influence on the distribution of anammox bacteria in Cape Fear River estuarine sediments [25]. *Scalindua* bacteria are ubiquitously distributed in marine ecosystems and are assumed to have a higher tolerance of salinity than *Kuenenia* and *Brocadia* bacteria [46]. However, Kartal et al. [47] showed that if the salt concentration increased gradually, *Kuenenia* bacteria could adapt to salt concentration as high as 30.0 ppt. In the present study, *Kuenenia* bacteria were ubiquitously distributed in the estuarine subtidal sediments within the salinity range of 11.9–25.2 ppt. Thus it was believed that *Kuenenia* bacteria also have the capability to cope with salinity. The discharge of large quantities of wastewater from the chemical industry zones into the Jiaojiang Estuary may have contributed to the salinity variation in the estuarine water column. Thus, it may be that the influence of salinity on the distribution of anammox communities in the subtidal sediments was also closely related to the intense chemical pollution in the Jiaojiang Estuary.

The overlying water column NO_3_^−^ concentration also contributed significantly to the spatial variations in the anammox communities (RDA results) and directly influenced the diversity and relative abundance of anammox bacteria (Pearson correlation analysis results) within the subtidal sediments of the Jiaojiang Estuary. The NO_3_^−^ concentration was also identified as an important factor influencing the distribution of anammox communities in estuarial mudflat sediment of the Mai Po Nature Reserve [41]. Ammonium and nitrite are the substrates of anammox bacteria. Ammonium is not a limiting factor for growth of anammox bacteria as it is always present in natural environments due to the degradation of organic matter. Very little or no nitrite could be measured because it's unstable in natural environments. As shown in Table 1, the concentration of nitrite was very low (less than 1 µM) in Jiaojiang estuarine subtidal sediments. It is likely that the nitrite concentration is the limiting factor for anammox bacteria in the estuarine subtidal sediments. Most of nitrite might be released by denitrifiers in the oxygen limited Jiaojiang estuarine subtidal sediments with relatively high nitrate concentration. Thus it is possible that the nitrate concentration influenced the production of nitrite and in turn influenced the communities (diversity and abundance) of anammox bacteria in the Jiaojiang Estuary. Estuaries are major conduits for the transport of anthropogenically derived inorganic nitrogen from the land to the sea. Indeed, various studies have shown that the wastewater treatment plants were the main sources of NO_3_^−^ in the estuarine water column [23], [27]. In the present study, the discharge of large quantities of chemical industry wastewater containing certain concentration of NO_3_^−^ provided a possible important source of NO_3_^−^ to the Jiaojiang estuarine water column [42]. Therefore, it is reasonable to speculate that the chemical pollution influenced the NO_3_^−^ concentration in the estuarine water column and, subsequently, influenced the distribution of anammox communities within the subtidal sediments.

This study provided the first direct evidence for the influence of chemical pollution on the community composition, diversity and abundance of anammox bacteria in the Jiaojiang Estuary of the East Sea (China). The nitrobenzene content and organic content of the estuarine sediment were found to have a substantial impact on the spatial distribution of anammox communities within the estuarine intertidal sediments. In contrast, the nitrobenzene content of the estuarine sediment, overlying water column NO_3_^−^ concentration and salinity appeared to be the most important environmental factors influencing the spatial distribution of anammox communities in the estuarine subtidal sediments.
